# Total Hip Arthroplasty Complications in Sickle Cell Disease: Systematic Review and Meta-Analysis

**DOI:** 10.3390/jcm13144129

**Published:** 2024-07-15

**Authors:** Fareed F. Alfaya, Ramy Mohamed Ghazy, Esraa Abdellatif Hammouda, Ahmed A. Mahfouz, Hamad Khalid Faya, Mohammed Abdulrahman M Asiri, Osama Hasan M. Alalmaie, Naif Yahya Alshahrani, Ali Zafer A Alqahtani, Abdulaziz Y. Alshahrani, Shaimaa Abdelaziz Abdelmoneim

**Affiliations:** 1 Department of Orthopedic Surgery, College of Medicine, King Khalid University, Abha 61421, Saudi Arabia; ffalfaia@kku.edu.sa; 2 Family and Community Medicine, College of Medicine, King Khalid University, Abha 61421, Saudi Arabia; mahfouz@kku.edu.sa; 3 Biomedical Informatics and Medical Statistics, Medical Research Institute, Alexandria University, Abha 61421, Egypt; hiph.eabdellatif@alexu.edu.eg; 4 Department of Clinical Research, El-Raml Pediatric Hospital, Ministry of Health and Population, Alexandria 21563, Egypt; 5 Department of Epidemiology, High Institute of Public Health, Alexandria University, Abha 61421, Egypt; 6 Faculty of Medicine, King Khalid University, Abha 61421, Saudi Arabia; a.e.7md.e.a@gmail.com (H.K.F.); mohammed5151.hot@gmail.com (M.A.M.A.); almaee.456@gmail.com (O.H.M.A.); naify.h507@gmail.com (N.Y.A.); dr.12ali@hotmail.com (A.Z.A.A.); azizwfdi@icloud.com (A.Y.A.); 7 Clinical Research Administration, Alexandria Directorate of Health Affairs, Egyptian Ministry of Health and Population, Alexandria 21554, Egypt

**Keywords:** sickle cell disease, total hip arthroplasty, complications, revision, crisis, prosthetic joint infection (PJI)

## Abstract

**Background**: Microvascular occlusions caused by sickle-shaped erythrocytes in patients with sickle cell disease (SCD) can lead to increased intraoperative and postoperative complications during total hip arthroplasty (THA). This systematic review and meta-analysis aimed to estimate the overall rate of complications following THA in patients with SCD and to identify the predictors of these complications including the surgical approach. **Methods**: The search was conducted across the grey literature, Google Scholar, and seven databases: Scopus, MEDLINE Central/PubMed, ProQuest, SciELO, SAGE, and Web of Science. All observational studies reporting the proportional THA complications in SCD were included. The Newcastle–Ottawa Scale quality assessment tool was used to assess the quality of the studies. The random effect model was applied to estimate the pooled outcomes. A sub-group analysis for the different approaches was performed. A sensitivity analysis and meta-regression were used to explain heterogeneity and to identify the THA complication predictors. **Results**: Of 3230 citations, only 23 studies were eligible for the meta-analysis. The pooled proportion of total primary THA complications in patients with SCD was 42% (95% CI: 30–56%, I^2^ = 95%). The sub-group analysis highlighted the anterolateral approach as the approach accompanied with the least complications. The meta-regression revealed that the anterolateral approach decreases the complications significantly, −28.67 (95%CI, −56.45–−0.88, *p* = 0.044), while the number of hips increased the complications by 0.43 (95%CI, 0.30–0.57, *p* < 0.001). Male gender, age, lateral approach, and HbSS non-significantly affect the THA complications in SCD 52.05, 0.18, 6.06, and 55.78, respectively. The pooled proportions for an SCD crisis 9% (95%CI, 5–14%, I^2^ = 61%), dislocation 4% (95%CI: 2–7%, I^2^ = 66%), aseptic loosening 12% (95%CI, 7–20%, I^2^ = 91%), revision 6% (3–11, I^2^ = 92%), heterotopic ossification 12% (95%CI, 3–35%, I^2^ = 95%), and prosthetic joint infection (PJI) 6% (95%CI, 3–11%, I2 = 92%). The most fitted model of meta-regression illustrated that HbSS significantly increases PJI, 0.05 (95%CI: 0.02–0.08, *p* = 0.009), and male gender and age non-significantly increase PJI, 2.28 (95%CI: −4.99–13.56, *p* = 0.311) and 0.001 (95%CI: −0.27–0.27, *p* = 0.990), respectively. Meanwhile, the anterolateral, lateral, and posterior approaches non-significantly decrease PJI, −3.55, −0.92, and −1.27, respectively. The pooled proportion for a sickle cell disease crisis after revision was 16% (95%CI: 6–36%, I^2^ = 0) and for aseptic loosening after revision, it was 24% (95%CI: 12–43%, I^2^ = 0). **Conclusions**: This study revealed the high rate of complications in patients with SCD and highlighted that the anterolateral approach was associated with the lowest rate of complications. Furthermore, this study illustrated that homozygous (HbSS) individuals are more susceptible to prosthetic joint infection.

## 1. Introduction

Total hip arthroplasty (THA) is a surgical treatment that is widely recognized for its ability to relieve pain and restore function in patients suffering from severe hip joint osteoarthritis. The operation entails replacing damaged hip joint components with prosthetic materials, which can greatly improve the quality of life for many patients. However, individuals with sickle cell disease (SCD) face unique and significant challenges when undergoing THA [1]. SCD is an autosomal recessive disease caused by a point mutation in the GAG to GTG in the sixth codon of the β (beta) hemoglobin beta gene (HBB) found on chromosome 11, characterized by the synthesis of defective hemoglobin, resulting in sickle-shaped erythrocytes. These malformed cells are prone to clogging the microvasculature, resulting in persistent hemolytic anemia. The mutation of HBB results in the production of three proteins from the three known types of patients with SCD; heterozygous hemoglobin SC (HbSC) and hemoglobin (HbS) beta thalassemia are mild forms of SCD present in 30% of patients. The homozygous hemoglobin sickle cell gene (HBSS) is the most common type. Individuals with such a mutation are at greater risk of bone osteonecrosis induced by microvascular blockage due to defective erythrocyte architecture [1,2].

Different surgical approaches are used in THA, including the posterior, lateral, and anterolateral approaches. The posterior approach involves an incision behind the hip, providing an excellent visualization of the acetabulum and femur. However, despite its advantages, it is associated with higher risks of infection and dislocation in patients with SCD. In the lateral approach (Hardinge approach), the incision splits the gluteus muscle from the side position, which lowers dislocation probabilities while being associated with delayed wound healing and nerve injury. The last approach is the anterolateral one. It depends on muscle-protective techniques to decline soft tissue damage and may lead to faster recovery; however, there is a higher probability of infection and thromboembolic events [3,4]. In addition, surgical techniques in implant fixation play a fundamental role in the long-term post-surgical safety of patients. Cemented fixation used in elderly patients with poor bone quality provides immediate and durable fixation, whereas cementless fixation depends on biological fixation in younger patients with higher physical activity and better bone quality. The hybrid technique involves cemented components of the acetabular (hip socket) components of the cementless femoral (thigh bone) components [5].

Microvascular occlusions caused by sickle-shaped erythrocytes in patients with SCD can lead to increased intraoperative and postoperative complications during THA. Wound complications, infection, periprosthetic fracture, aseptic loosening, and revision increased in patients with SCD who underwent THA [6]. One important issue is the elevated risk of infections. The compromised vasculature and immune function in patients with SCD make them more susceptible to bacterial infections, which can potentially jeopardize the success of the surgery [7]. Given the increased risks and difficulties associated with THA in patients with SCD, a multidisciplinary approach is required to optimize outcomes. Intraoperative techniques should prioritize limiting blood loss, good hydration, maintaining adequate oxygenation, and closely monitoring symptoms of thromboembolism. Postoperative care must prioritize infection avoidance, meticulous wound care, and close monitoring of thromboembolic events. Pain management regimens should be adapted to specific needs of patients with SCD, balancing effective pain relief with the prevention of opioid-related comorbidities [8].

Although THA can offer significant benefits to patients with severe hip joint disease, individuals with SCD face unique and challenging obstacles. Identifying the specific risks and obstacles that patients with SCD confront during hip arthroplasty is critical for improving perioperative treatment and, ultimately, patient outcomes. As a result, healthcare practitioners should prioritize avoiding and managing these complications early to minimize their negative impact on patients’ rehabilitation and quality of life.

## 2. Methods

**Figure 1:**
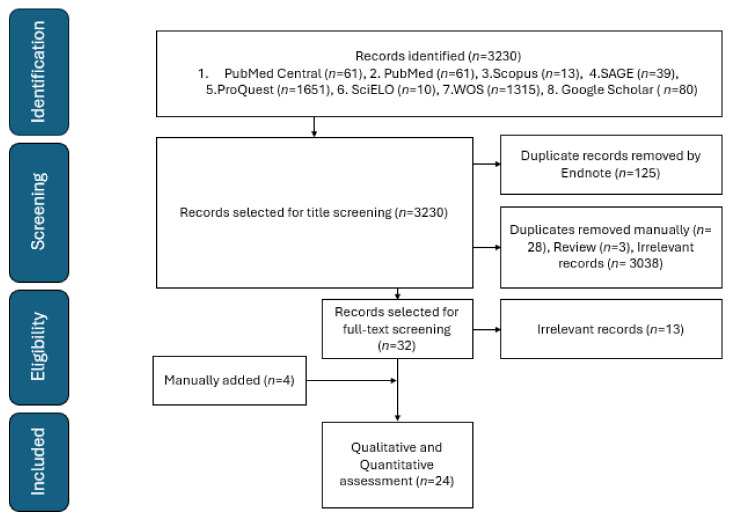
PRISMA flowchart of included studies.

This study measures the pooled proportion of primary, revision THA complications in SCD, and identifies the predictors for these complications including the surgical approach. This meta-analysis follows the recommendations of the Preferred Reporting Items for Systematic Reviews and Meta-Analyses (PRISMA) and Cochrane Handbook for Systematic Reviews of Interventions 2023 [9]. The search process included both the grey literature and published studies from the databases Scopus, MEDLINE Central/PubMed, ProQuest, SciELO, SAGE, Web of Science, and Google Scholar, covering the period from inception to 28 February 2024. The used search strategy is presented in Supplementary S1. All observational studies reporting the proportional THA complications in SCD were included with no date or language restriction. Studies reported only for a sickle cell trait, abstract-only papers, proposals, conference proceedings, editorials, reviews, case reports, case series, books, and duplicate records were excluded. All articles were imported into EndNote X9 to detect and remove duplicates. The non-duplicated studies were imported into a Microsoft Excel sheet containing the authors’ names, publication year, journal name, digital object identifier (DOI), URL link, and abstract. The titles and the abstracts were screened for eligibility by MAA and OHM, followed by full-text screening to identify the eligible articles (by YA and ADA). Screening was performed independently by two authors for each screening step. The senior author (SAA) solved any disagreement. A manual search for eligible studies was conducted by examining the references of the included studies. Figure 1 is the flowchart.

The extraction was performed by two authors (HKF, AYA) with the following predefined data: publication year, authors’ names, country, study design, study population characteristics, sample size, number of hips, duration of the study, inclusion and exclusion criteria, the total complications, detailed complications, for primary and revision THA, and surgical approaches. The review protocol was registered at PROSPERO (registration: CRD42024498437). Cochrane’s Q test (I^2^) was used to assess and measure heterogeneity between studies. Publications’ bias was determined by the visual inspection of the funnel plot and statistically by Egger’s test for each outcome. The Newcastle–Ottawa Scale quality assessment tool for non-randomized studies was used [10]. The quality score was either good (7–10 points), fair (3–6 points), or poor (0–2 points). The assessment was performed by two independent reviewers (EAH and SAA).

The primary THA is a surgical procedure in which the worn out or damaged parts are removed and replaced with artificial components, “prostheses or implants”, that are made of metal with joint surfaces covered with polyethylene or ceramic. Revision hip arthroplasty is a replacement of all the implants that were inserted before in the primary total hip arthroplasty. This procedure is performed when the primary implant starts to wear out or becomes infected [11].

The primary outcome of this study is to determine the pooled proportion of complications following primary THA, including overall complications, prosthetic joint infection (PJI), SCD crises, aseptic loosening, revision surgeries, dislocations, and heterotopic ossification. Additionally, we examined complications following revision surgeries, specifically SCD crises and aseptic loosening. The secondary outcome was to identify predictors of overall complications and to determine which surgical approach is associated with the fewest complications.

### Statistical Analysis

The statistical analyses were conducted using R 4.3.2 software. The publication bias was assessed for the overall complications and each type of complication through visual inspection of the funnel plot, Egger’s test for each outcome includes at least ten studies, and Beggs’ test for each outcome includes less than ten studies. The random effect models were used to illustrate the pooled proportions with substantial heterogeneity between the studies (I^2^ > 50), and the fixed effect models were used for those with low heterogeneity. To explain the substantial heterogeneity between the studies reporting the primary THA complications, a sensitivity analysis was performed using the leave-one-out analysis to identify and remove the influential studies and to adjust the pooled proportion, as well as the outliers removed from some types of complications (Figures S2 and S3). A sub-group analysis was conducted for the total THA complications according to the type of surgical approach to decide which approach had fewer complications. A meta-regression analysis was conducted to explain the heterogeneity between the studies when all previous steps could not explain the heterogeneity. The most fitted meta-regression model was selected based on the lowest Akaike Information Criterion (AIC), considering the complication pooled proportions as the dependent variable, and used to identify the predictors of the overall complications and PJI.

## 3. Results

### 3.1. Characteristics of the Studies

Twenty-four studies reported complications after primary and revision THA in 3016 hips in 3291 patients with SCD (Table 1). The mean age across the twenty studies was 29.70 ± 6.02 years. Nine studies were conducted in the United States of America (USA) [12,13,14,15,16,17,18,19,20], five from the Kingdom of Saudi Arabia (KSA) [21,22,23,24,25], two from Bahrain [5,26], two from the United Kingdom (UK) [27,28], one from Senegal [29], one from Nigeria [30], one from France [1], one from Yemen [31], one from Turkey [32], and one from India [33]. According to the Newcastle–Ottawa quality score, four studies were good, and twenty studies were fair. Three studies did not report the total complication and could not be estimated due to recurrent complications for the same patients [13,17,33].

### 3.2. Risk of Bias

**Figure 2:**
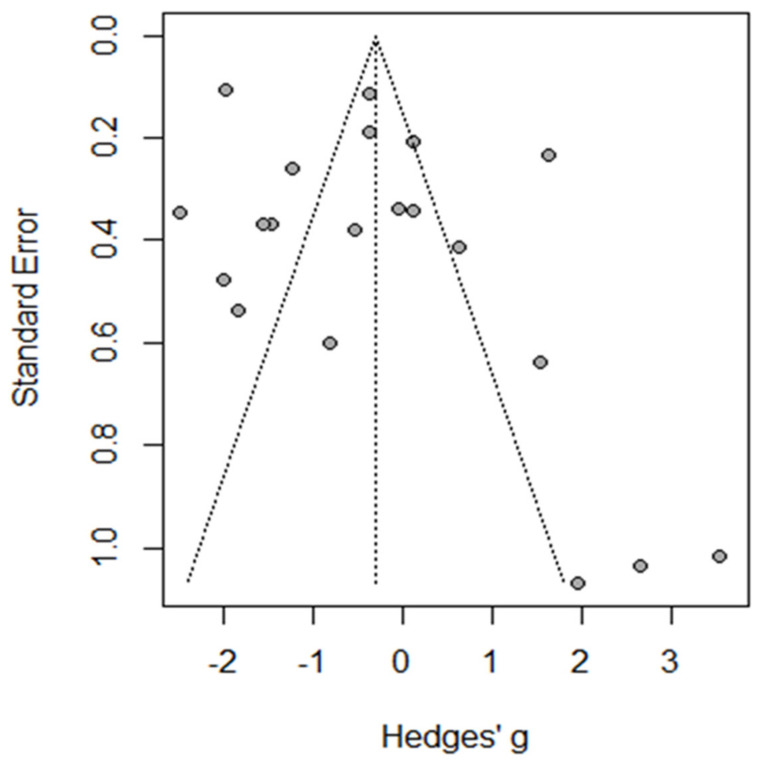
Funnel plot of total complications.

The funnel plot and Egger’s test indicated the absence of heterogeneity, with symmetric results for the funnel plot and non-significant results for Egger’s test of 1.45 (95%CI: −0.87–−5.9, *p* = 0.161) (Figure 2).

### 3.3. The Primary THA Complications

#### 3.3.1. The Pooled Proportions of the Total Complications

**Figure 3:**
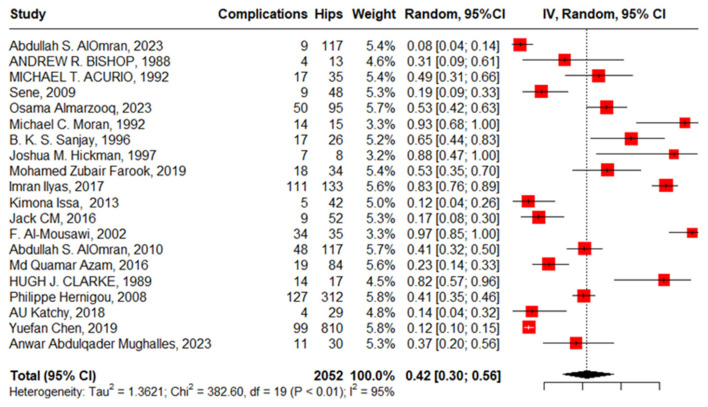
Forest plot of primary THA total complications in SCD.

**Figure 4:**
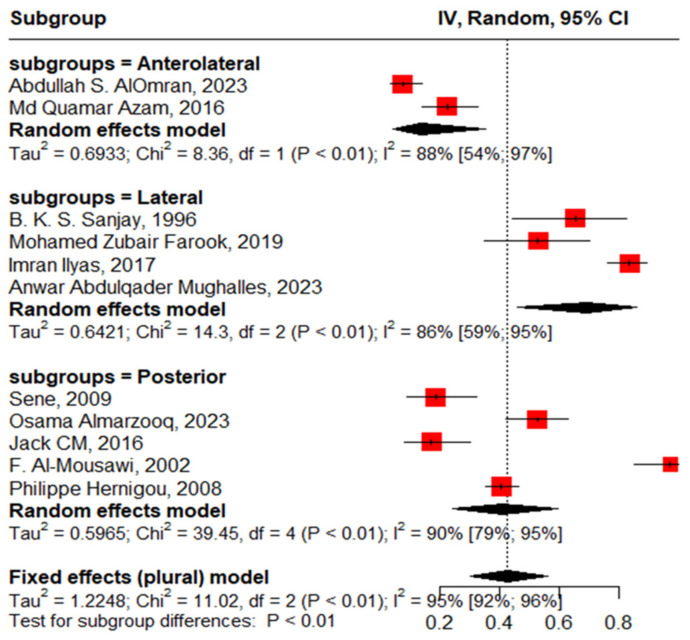
Sub-group analysis according to surgical approach.

**Figure 5:**
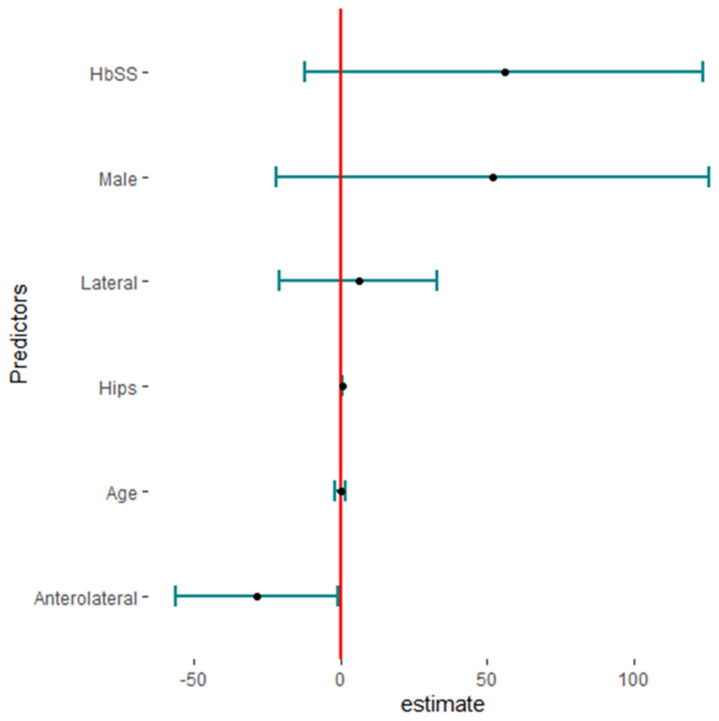
The predictors of total complications in primary total hip arthroplasty in sickle cell disease (HBSS: homozygous HbS disease, Hips: number of hips).

Twenty studies reported complications in 2052 hips in 1788 patients with SCD after primary THA. The pooled proportion of total complications was 42% (95% CI, 30–56%, I^2^ = 95%), ranging between 8% (95%CI, 4–14%) reported by Abdullah S. Alomran, 2023 [22], and 97% (95%CI, 85–100%) reported by F. Al-Mousawi, 2002 [26] (Figure 3). The sub-group analysis for eleven studies according to the used surgical approaches revealed substantial variations in complications across the different surgical approaches (*p* < 0.01) with a pooled proportion of 43% (95%, 30–56%, I^2^ = 95%), where the anterolateral approach was associated with the least complications, 14% (95%, 4–35%, I^2^ = 88%); posterior approach, 41% (95%, 24–60%, I^2^ = 90); and lateral approach, 69% (95%, 46–86%, I^2^ = 86%) (Figure 4). The leave-one-out sensitivity analysis identified Yuefan Chen’s work, 2019 [15], as an influential study, and the heterogeneity decreased only to I^2^ = 91%, and a pooled proportion of 44% (95%CI, 32–56%) suggests that the pooled effect size is relatively stable across different subsets of studies. “Abdullah S. AlOmran, 2023”, “Michael C. Moran, 1992”, “Imran Ilyas, 2017”, “Kimona Issa, 2013”, “F. Al-Mousawi, 2002”, “HUGH J. CLARKE, 1989”, and “Yuefan Chen, 2019” [15,16,19,20,22,24,26] were identified as outliers and heterogeneity decreased to 79% after assigning a weight of zero to the identified outliers and the pooled proportion decreased to 38% (95%CI, 30–46%). Meta-regression explained 45% of heterogeneity with residual heterogeneity tau2 = 0.6310 (SE = 0.6917) and referred heterogeneity significantly to homozygous sickle hemoglobin (SS), 11.74 (95%CI, 4.47–19.02%, *p* = 0.001); mean age, 0.72 (95%CI, 0.30–1.13, *p* < 0.001); male gender, 55.33 (95%CI, 22.83–87.82, *p* < 0.001); lateral approach, −1.98 (95%CI, −3.96–−0.01, *p* = 0.048); number of hips, −0.008 (95%CI, −0.01–−0.001, *p* = 0.036); and the interaction term of mean age and male gender, −1.52 (95%CI, −2.42–−0.62, *p* = 0.001), where the posterior approach, anterolateral approach, and cemented/cementless surgery did not significantly contribute to heterogeneity, 0.13 (95%CI, −1.95–2.22, *p* = 0.897), −1.01 (95%CI, −3.11–1.09, *p* = 0.346), and 2.46 (95%CI: −0.83–5.76, *p* = 0.142), respectively. The most fitted model revealed that the anterolateral approach significantly decreased the THA complications in SCD, −28.67 (95%CI, −56.45–−0.88, *p* = 0.044), while the number of hips increased the complications by 0.43 (95%CI, 0.30–0.57, *p* < 0.001). Male gender, age, lateral approach, and HbSS did not significantly affect the THA complications in SCD, 52.05 (95%CI, −21.77–125.87, *p* = 0.142), −0.18 (95%CI, −2.20–1.82, *p* = 0.835), 6.06 (95%CI, −20.98–33.10, *p* = 0.619), and 55.78 (95%CI, −12.22–123.79, *p* = 0.095), respectively (Figure 5).

#### 3.3.2. SCD Crisis

**Figure 6:**
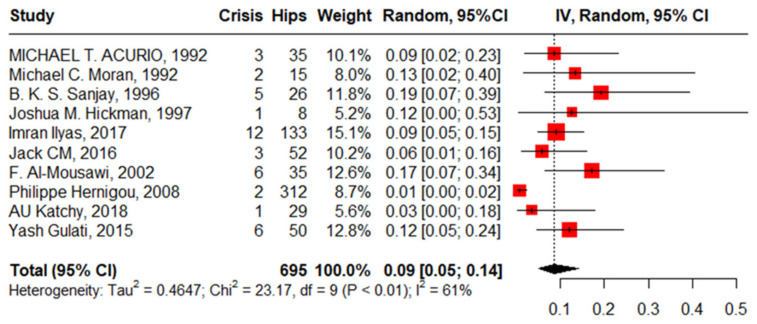
Forest plot of pooled proportion of sickle cell disease crisis after primary total hip arthroplasty.

Ten studies reported an SCD crisis after primary THA in 695 hips in 544 patients with SCD with a pooled proportion of 9% (95%CI, 5–14%, I^2^ = 61%), ranging between 1% (95%CI, 0–2%) reported by Philippe Hemigou, 2008 [1], and 19% (95%CI, 7–39%) reported by Sanjay, 1996. The funnel plot and Egger’s test, −0.93 (−4.17–1.47, *p* = 0.375), illustrated the absence of publication bias. The leave-one-out sensitivity analysis identified Philippe Hernigou’s work, 2008 [24], as an influential study, and the heterogeneity decreased to I^2^ = 0% and the pooled proportion was 11% (95%CI, 8–15%) (Figure 6).

#### 3.3.3. Dislocation after Primary THA

**Figure 7:**
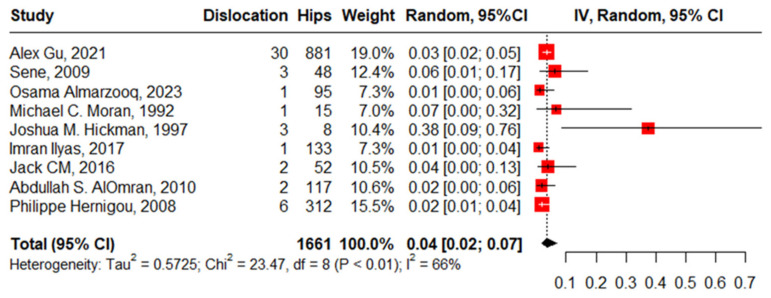
Forest plot of proportion of dislocation after primary total hip arthroplasty.

Nine studies reported dislocation in 1661 hips in 1490 patients with SCD and the pooled proportion with moderate heterogeneity was 4% (95%CI: 2–7%, I^2^ = 66%), ranging between Osama Almarzooq’s work, 2023 [5], at 1% (95%CI: 0–6%) and Joshua M. Hickman’s work, 1997 [18], at 38% (95%CI: 9–76%). The funnel plot and significant Beggs’ test (*p* < 0.001) declared the existence of publication bias. After removing the outlier, regarding Joshua M. Hickman’s work, 1997 [18], the pooled proportion decreased to 3% (95%CI: 2–4%, I^2^ = 11%) (Figure 7).

#### 3.3.4. Aseptic Loosening

**Figure 8:**
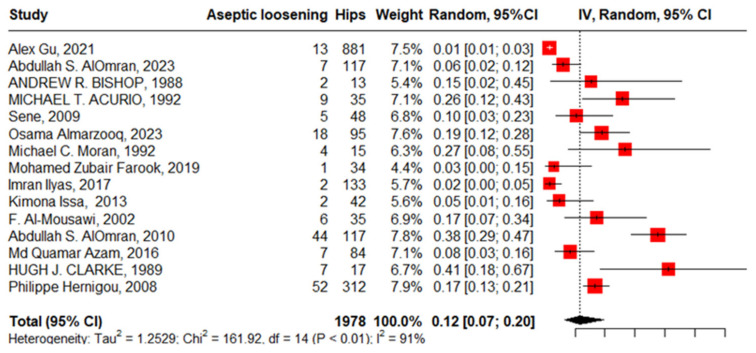
Forest plot of pooled proportion of aseptic loosening after primary total hip arthroplasty.

Fifteen studies reported aseptic loosening with 1978 hips in 1739 patients with SCD; the pooled proportion represented with high heterogeneity was 12% (95%CI, 7–20%, I^2^ = 91%), ranging between Alex Gu’s work, 2021 [17], at 1% (95%CI, 1–3%) and Clarcke’s work, 1989 [16], at 41% (95% CI, 18–67%) (Figure 8).

The funnel plot and non-significant Egger’s test, −1.13 (95%CI, −5.49–1.46, *p* = 0.275), declared the absence of publication bias. “Alex Gu, 2021”, “Imran Ilyas, 2017”, “Abdullah S. AlOmran, 2010”, and “Clarke, 1989” [16,17,21,24] were identified as outliers where the heterogeneity improved from 91% to 57% after being weighted to zero, and the pooled proportion increased to 13% (95%CI, 10–18%).

#### 3.3.5. PJI after Primary THA

**Figure 9:**
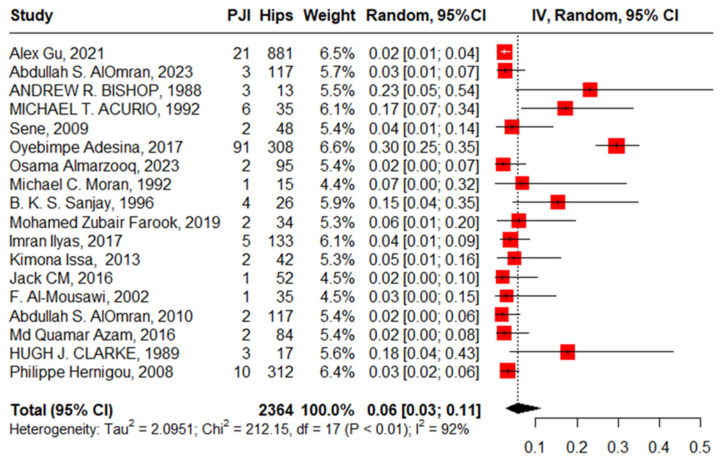
Forest plot of pooled prosthetic joint infection after primary total hip arthroplasty.

**Figure 10:**
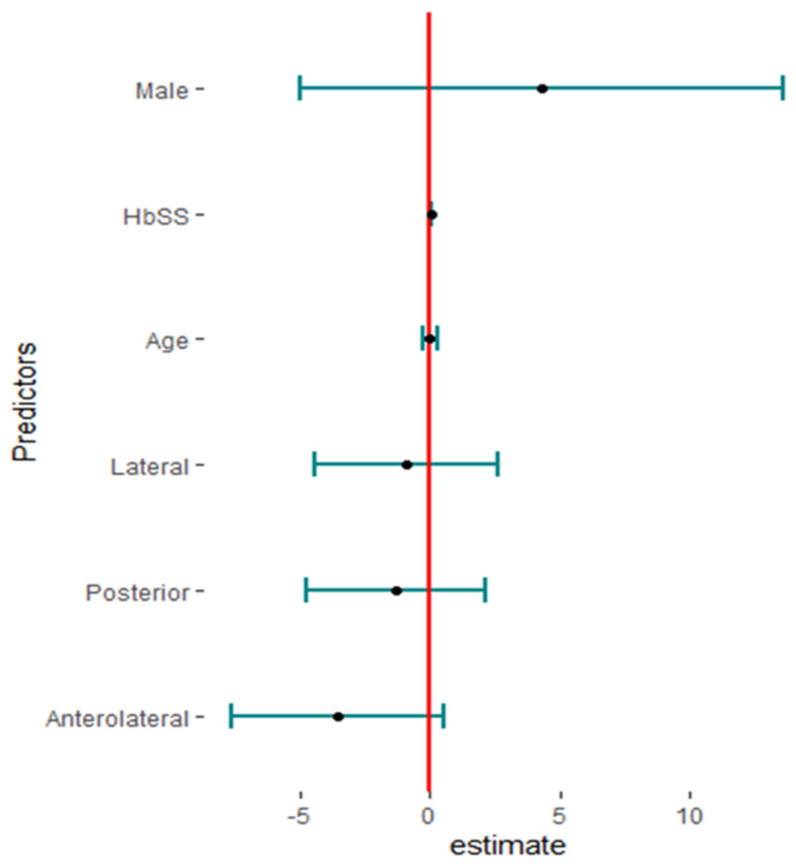
Fitting model of meta-regression of prosthetic joint infection.

Eighteen studies reported PJI after primary THA with 2364 hips in 2108 patients with SCD; the pooled proportion was 6% (95%CI, 3–11%, I^2^ = 92%), ranging between Jack C M’s work, 2016 [28], at 2% (95%CI, 0–10%) and Oyebimpe Adesina’s work, 2017 [13], with 30% (95%CI, 25–35%) (Figure 9). The funnel plot and significant Egger’s test, −2.575 (−5.3–−0.72, *p* = 0.020) highlight the presence of publication bias. The leave-one-out sensitivity analysis revealed that Oyebimpe Adesina’s work, 2017 [13], was an influential study, where the heterogeneity improved to 66% after removing it and the pooled proportion decreased to 4% (95% CI, 3–5%). Meta-regression explained 100% of the heterogeneity with tau^2^ = (SE = 0.2777) in terms of age, male gender, anterolateral approach, lateral approach, posterior approach, number of hips in each study, and the quality of the studies, where HbSS significantly drives the heterogeneity between the studies, −2.90 (95%CI: −5.646–−0.159). The most fitted model with R^2^ = 70.62% illustrated that HbSS increased PJI with 0.05 (95%CI, 0.02–0.08, *p* = 0.009); anterolateral approach, −3.55 (95%CI, −7.60–0.50, *p* = 0.077). Meanwhile, male gender increases PJI non-significantly, 2.28 (95%CI, −4.99–13.56, *p* = 0.311); lateral approach, −0.92 (95%CI, −4.45–2.61, *p* = 0.558); and age, 0.001 (95%CI, −0.27–0.27, *p* = 0.990), where the posterior approach decreases PJI non-significantly, −1.27 (95%CI, −4.71–2.17, *p* = 0.412). Notably, the lateral approach had the non-significant highest complication rate, while the anterolateral approach had the non-significant lowest complication rate (Figure 10).

PJI-causative organisms were reported by nine studies [1,12,14,20,22,24,28,29,32], where *Staphylococcus aureus* and *Staphylococcus epidermidis* were the most prevalent organisms reported by these studies (Table 2).

#### 3.3.6. Heterotopic Ossification

**Figure 11:**
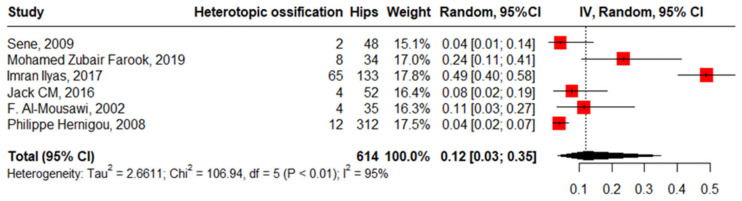
Forest plot of pooled proportion of heterotopic ossification.

Six studies reported the pooled heterotopic ossification in 614 hips among 481 patients at 12% with high heterogeneity (95%CI, 3–35%, I^2^ = 95%), ranging between 4% (95%CI, 1–14%) and 49% (95%CI, 40–58%). The funnel plot and insignificant Begg’s test (*p* = 0.136) revealed the absence of publication bias. Meta-regression showed that HbSS and the lateral approach drive the heterogeneity significantly, 3.47 (95%CI, 0.448–6.503) and 1.75 (95%CI, 0.741–2.764), respectively. Meanwhile, male gender increases heterogeneity non-significantly, 1.68 (95%CI, −5.254–8.634) (Figure 11).

#### 3.3.7. Revision after Primary THA

**Figure 12:**
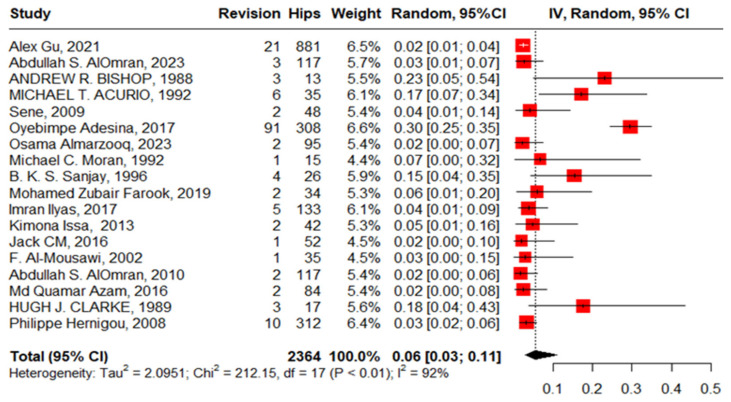
Forest plot of pooled proportion of revision after primary total hip arthroplasty.

Eighteen studies reported revision in 2364 hips in 1811 patients with SCD with high heterogeneity, 6% (3–11, I^2^ = 92%), ranging between 2% (95%CI: 0–10%) in Jack CM’s work, 2016, and 23% (95%CI: 5–54%) in Andrew r. Bishop’s work, 1988. The publication bias and non-significant Egger’s test revealed the absence of publication bias, 0.206 (95%CI: −3.04–3.75, *p* = 0.839). “Alex Gu, 2021”, “Acurio, 1992”, “Imran Ilyas, 2017”, “Abdullah S. AlOmran, 2010”, and “Clarke, 1989” [12,16,17,21,24] were outliers studies and after assigning a weight of zero to the identified outliers, the heterogeneity decreased to 58% and the pooled proportion increased to 14% (95%CI, 11–19%) (Figure 12).

## 4. Complications after Revision

### 4.1. SCD Crisis after Revision

**Figure 13:**
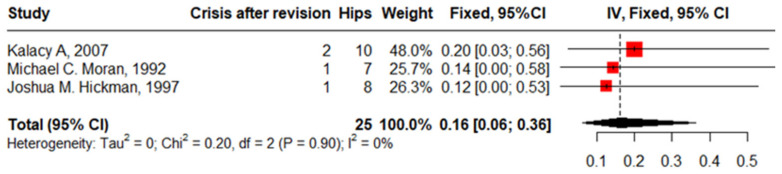
Forest plot of sickle cell disease crisis after revision.

Three studies reported a crisis in 25 hips in 25 patients with SCD after revision with zero heterogeneity, 16% (95%CI, 6–36%, I^2^ = 0). The funnel plot and non-significant Begg’s test (*p* = 1.0) illustrated the absence of publication bias (Figure 13).

### 4.2. Aseptic Loosening after Revision

**Figure 14:**
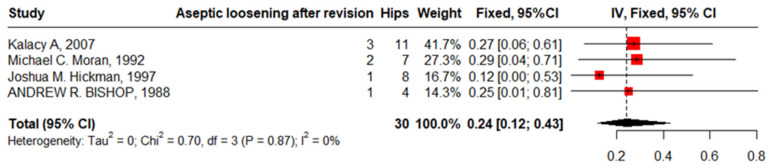
Forest plot of pooled proportion of aseptic loosening after revision.

Four studies reported aseptic loosening after revision in 30 hips in patients with SCD with zero heterogeneity of 24% (95%CI: 12–43%, I^2^ = 0). The funnel plot and non-significant Begg’s test (*p* = 0.333) revealed nonpublication bias (Figure 14).

## 5. Discussion

SCD is a prevalent hereditary disorder that leads to osteonecrosis of the femoral head as a common complication in patients with SCD, often requiring THA. However, the literature suggests that THA in patients with SCD is associated with a significantly higher risk of postoperative complications compared to the general population. This meta-analysis aimed to assess the postoperative complications of both primary and revision surgeries of THA in SCD to provide healthcare professionals guidelines to manage the pre-, intra-, and postoperative period to reduce the postoperative complications. The studies included 24 observational studies on 3016 hips in 3291 patients with SCD.

Total complications were reported in 2052 hips in 1788 patients. The pooled proportion of total complications was 42% (95%CI: 30–56%). This proportion was higher than what was documented in recent meta-analyses, which ranged from 14 to 16% [6,34]. The difference in complication classification methods between the current and previous studies might explain the higher proportion observed. Previous studies divided complications into medical and surgical complications, whereas we counted them collectively. The result showed an association of complications with the surgical approaches where the anterolateral approach significantly demonstrated the lowest proportion of complications compared to the two other approaches. Ohta et al. [35] reported that the anterolateral approach keeps the posterior capsule and lowers the risk of postoperative dislocation compared to the posterior one. Additionally, the current study reported that HbSS, age, and male gender were associated with a non-significant increased proportion of complications. These findings were in line with the literature where HbSS has a lower oxygen affinity, which maximizes its need for a preoperative blood transfusion to target Hb of 10 g/dL. [36] However, the risk of developing red cell alloantibodies, iron load, and hemolytic transfusion reaction was observed particularly in aggressive transfusion [37,38]. Likewise, Fassihi et al. [6] noticed that complications were more prevalent in younger patients and could be attributed to the stress placed at the implant–bone interface, which can lead to implant failure.

Crises and aseptic loosening were documented as complications after both primary and revision surgeries. Crises had a pooled proportion of 9%. This proportion was much higher than documented by Fassihi et al. [6] (7.5%). Previous studies attributed crises to multiple preoperative triggers (hypoxia, infection, dehydration, increased blood viscosity, and emotional stress) [39]. Further research is required to investigate the impact of adjusting these triggers on the prevalence of a crisis. Aseptic loosening known as a failure of the fixation of a prosthetic component in the absence of infections was documented in 15 studies with a pooled proportion of 12% (7–20%).

Eighteen studies involved PJIs after primary THA in 2364 hips in 2108 patients with SCD. The most common organisms detected were “Staphylococcus Aurus”, “Staphylococcus Epidermidis”, “Acinetobacter Calcoaceticus”, “Proteus Vulgaris”, and “Pseudomonas Herrelea”. The infection proportion increased for those who have HbSS versus HbS, which aligns with previous studies’ findings [20]. Patients with SCD are susceptible to infections due to functional asplenia, compromised immunity, reduced blood flow through bone caused by sickling, and frequent intravenous procedures required for treating their primary condition. The formation of wound drainage and hematomas also elevates the infection risk. Additionally, extended surgical durations resulting from technical challenges further amplify this risk [25]. Loosening and infection are the most common complications in patients with SCD. In this meta-analysis, patients with SCD who underwent the anterolateral approach had fewer complications compared to the other approaches. These results support Butler et al.’s [40] findings of a higher infection rate in the posterolateral approach (5.5%) compared to the direct lateral approach (4.2%), and anterolateral approach (2.4%) in non-SCD patients. This remarkable finding requires additional research to elucidate its clinical interpretation. Eighteen studies documented revision after primary THA in 2364 hips in 1011 patients with SCD with a pooled prevalence of 6% (95%CI: 3–11). This wide range was due to the presence of outlier studies [12,16,21,24]. The timing of the conducting of studies could contribute to the wide range of the proportion of revisions, as Jack et al. [28] reported a proportion of 2% in cases followed up from 2002 to 2011, while Bishop et al.’s study [14] (23% revision) followed cases from 1974 to 1984. This variation in the percentage of revision cases may be attributed to the differences in surgical techniques and technologies applied in the different time periods. Four studies reported complications after revision that were mostly an SCD crisis and aseptic loosening, although the pooled proportion of crises after the primary THA is 9% and that of loosening is 12%; after revision, crises increased to 16% and loosening to 24%. These results agree with the previous studies [23].

### Strengths and Limitations

Our study has several strengths and limitations. The first strength is that our study is the first meta-analysis to discuss THA complications in SCD with many types of complications after primary and revision THA. The second strength is the wide sensitive search strategy in different databases. Third, most of the included studies were of moderate to high quality. However, our limitation is that we could not identify the determinants of all the detected complications, especially blood transfusion complications as reported in many ways in the studies. Additionally, the analysis was limited by the quality of the original studies and found heterogeneity and publication bias as in the analysis of dislocation. Caution should be used when interpreting these results. Finally, the included studies were conducted over several decades (1988–2024), which made variations in the surgical techniques and instruments used from recent studies.

In conclusion, this meta-analysis reveals that patients with SCD undergoing THA experience a significantly higher rate of postoperative complications, with a pooled complication rate of 42%. Notably, the anterolateral surgical approach is associated with fewer complications. More studies are needed to identify more predictors of THA complications in patients with SCD.

## Figures and Tables

**Table 1:** Characteristics of studies included in the systematic review.

Author, Date, Country	Sample Size/Hips	Age (yrs), Male %	Population Criteria	Primary/Revision	Cement/Approach Type/SS%	Total Complications/Hips n (%)	Revision as Complication n (%)	Crisis n (%)	PJI n (%)	Aseptic Loosening n (%)	Dislocation n (%)	Heterotopic Ossification n (%)	Others	Follow-Up (year)	Quality Score
Alex Gu, [17] 2021, USA	881/881	NA 40%	SCD + osteonecrosis, Follow-up >2 yrs, Unilateral, >18 yrs	Primary	NA NA NA	NA	35 (3.97)	-	21 (2.38)	13 (1.47)	30 (3.40)	0	Blood loss: 340, DVT: 136, HF: 155, PE: 125, Respiratory: 133, UTI: 252, Sepsis: 241, Stroke: 77, Pneumonia: 133	2 years	7
AlOmran, [22] 2023, KSA	92/117	22 36%	SCD underwent THA, Follow-up >4 yrs	Primary	0 (0%), Anterolateral 76 (82%)	9/117 (8)	7 (5.98)	-	3 (2.56)	7 (5.98)	-	-	Osteolysis: 1, Skin infection: 3	12.4 (4–16).	5
Bishop, [14] 1988, USA	11/13	30.7 46%	Confirmed SS, SC, or S-thalassemia, at Grady Hospital, 1974–1984	Primary	15 (89%), Posterior or direct lateral 6 (54.5%)	4/13 (31)	1 (7.69)	-	3 (23.07)	2 (15.38)	-	-	Fracture: 1, Resection: 3,	7.5 (2–13)	6
Acurio, [12] 1992, USA	25/35	30 60%	Osteonecrosis secondary to SC haemoglobinopathy, >18 yrs	Primary	17 (48%) NA 11 (44%)	17/35 (48)	14 (40.0)	3 (8.57)	6 (17.14)	9 (25.71)	0	0	Skin infection: 1, Blood loss: 2, Resection: 9, Fracture: 2, Osteolysis: 9, UTI: 3	8.6 (2–18)	5
Sene, [29] 2009, Senegal	38/48	22 29%	Posterolateral approach	Primary	48 (100%) Posterolateral 26 (68.4%)	9/48 (19)	12 (25)	0	2 (4.16)	5 (10.41)	3 (6.25)	2 (4.16)	Osteolysis: 3, Fracture: 1	5 years	5
Adesina, [13] 2017, USA	308/308	36 47.4%	High probability of SCD, performed SCD hip surgery after 1st ONFH, age < 65	Primary	NA NA NA	NA	0	193 (62.66)	91 (29.54)	0	0	0	DVT: 4	1–3 mon.	8
Almarzooq, [5] 2023, Bahrain	69/95	NA 47%		Primary	40 (42%) Posterior NA	50/95 (53)	22 (23.15)	-	2 (2.10)	18 (18.94)	1 (1.05)	0	DVT: 1, Osteolysis: 17, Small particle disease: 5, Fracture: 2	NA	6
Moran, [20] 1992, USA	14/15	37 36%	End-stage osteonecrosis of femoral head secondary to SCH after primary and revision THA	Primary	13 (86%), Posterior: 14% Lateral: 86% 11 (78.5%)	14/15 (93)	5 (33.33)	2 (13.33)	1 (6.66)	4 (26.66)	2 (13.33)	0	DVT: 1, HF: 2, UTI: 1, Sepsis: 1 Transfusion reaction: 2, Wound drained: 3, Perforation: 2, Hematoma: 2	4.8 years	5
Sanjay, [25] 1995, KSA	21/26	26.9 42.9%	Avascular necrosis of femoral head in SCD from 1987–1992	Primary	0 (0%), Lateral 20 (95.2%)	17/26 (65)	2 (7.69)	5 (19.23)	4 (15.38)	0	0	0	Hematoma: 3, Fracture: 5	4.6 years	5
Hickman, [18] 1997, USA	11/8	40 36.4%	1° HA with SCD	Primary	1 (12.5%), Posterolateral: 81.25% Lateral: 18.75% 7 (63%)	7/8 (90)	2 (25)	1 (12.50)	0	0	3 (37.50)	0	DVT: 1, Hematoma: 1, Femoral perforation: 1, Blood loss: 2	6 (2–12)	6
Farook, [27] 2019, UK	30/34	36.7 40%	Grade IV osteonecrosis in femoral head + 2° hip OA	Primary	3 (27.8%) Lateral 23 (77%)	18/34 (53)	6 (17.64)	0	2 (5.88)	1 (2.94)	0	8 (23.52)	PE: 1, Osteolysis: 4, Eccentric polyethylene wear: 2, Femoral shaft perforation: 2	10.5 (1–18)	6
Ilyas, [24] 2017, KSA	101/133	25 48.5%	SCD with THA, SS, arthritis secondary to AVN ≥ Steinberg grade IV, No bipolar cups	Primary	0 (0%) Lateral 101 (100%)	111/133 (83)	5 (3.75)	12 (9.02)	5 (3.75)	2 (1.50)	1 (0.75)	65 (48.87)	Resp.: 3, Osteolysis: 7, Skin infection: 4, Fracture: 6	14.59 (5–17)	5
Issa, [19] 2013, USA	32/42	37 86.8%	THA due to osteonecrosis, Follow-up > 3 yrs	Primary	0 (0%) Anterolateral 37 (88.1%) Posterior: 5 (11.91%) 20 (62%)	5/42 (12)	5 (11.90)	0	2 (4.76)	2 (4.76)	0	0	Femoral head change: 1	7.5 (5–11)	6
Jack CM, [28] 2016, UK	40/52	36.1 38.5%	SCD performed, cementless	Primary	0 (0%) Posterior NA	9/52 (17)	0	3 (5.76)	0	0	0	2 (3.84)	Respiratory: 2, UTI: 1, Sepsis: 1, Chicken pox: 1	5.2 (2–10.1)	4
Al-Mousawi, [26] 2002, Bahrain	28/35	27.5 53.6%	SCD with AVN of femoral head underwent THA	Primary	NA Posterolateral 27 (96.4%)	34/35 (97)	6 (17.14)	6 (17.14)	1 (2.85)	6 (17.14)	0	4 (11.42)	Blood loss: 2	9.5 (5–15)	5
AlOmran, [21] 2010, KSA	118/136	NA 19.1%	D9 grade III Ficat, >ANFH and cementless porous-coated proximal femur fixation	Primary	46 (33%) NA 97 (82%)	48/136 (35)	48 (35.29)	0	2 (1.47)	44 (32.35)	2 (1.47)	0		(2–16)	6
Azam, [23] 2016, KSA	67/84	24 55.2%	Grade IV, osteonecrosis of femoral head	Primary	0%, Anterolateral, 39 (58.2%)	19/84 (23)	8 (9.52)	0	2 (2.38)	7 (8.33)	0	0	Respiratory: 2 Hematoma: 7 Superficial infection: 6	7.5 (4–12)	5
Clarke, [16] 1989, USA	15/27	33 73.3%	SCD with avascular necrosis of the femoral head	Primary 17/27 (41.17%)	13 (76.4%), Posterolateral: 81.48% Lateral: 18.51% 7 (46.6%)	14/27 (52)	10 (37.03)	0	3 (11.11)	7 (25.92)	0	0	Fracture: 4	2 years	4
Hernigou, [1] 2008, France	244/312	32 48.4%	SCD underwent surgery from 1980 to 2000 with osteonecrosis, Hips ≥ stage IV by Steinberg classification	Primary	0 (0%) Posterolateral 145 (59.4%)	127/312 (41)	42 (13.46)	2 (0.64)	10 (3.20)	52 (16.66)	6 (1.92)	12 (3.84)	DVT: 1, PE: 1, Respiratory: 4, Transfusion reaction: 71, Femoral perforation: 6, Repeat fixation of the cup: 4, Peroneal nerve palsy: 2, Isolated acetabular wear: 3, Hematoma: 4	13 (5–25)	6
AU Katchy, [30] 2018, Nigeria	21/29	23.8 86%	AVN of femur head secondary to SCD	Primary	0 (0%) Anterolateral	4/29 (14)	0	1 (3.44)	0	0	0	0	Blood transfusion: 1, Resp.: 1, Fracture: 1	(1–5)	4
Chen, [15] 2019, USA	810/810	66.5 39%	Complete records, ≥18 years	Primary	NA	99/810 (12)	0	0	0	0	0	0	HF: 11, Respiratory: 37, Intraoperative: <11, GIT: <11, Infections: 26	0.25	7
Gulati, [33] 2015, India	39/50	22 28%	Steinberg III–V	Primary	0 (0%) Lateral 19 (48%)	NA	0	6 (12.0)	0	0	0	0	Blood transfusion: 5, Leg length discrepancy: 3, Hematoma: 3, Wound discargepersistant: 2, Fracture: 1	3.8 (2–6)	3
Mughalles A [31], 2024, Yemen	27/30	27 74%	SCD, grade IV–VI osteonecrosis of femoral head, 2° hip OA	Primary	0 (0%) Lateral 27 (100%)	11/30 (37)	0	0	0	0	0	0	Respiratory: 2 Leg length discrepancy: 30 Fractures: 5 Superficial infections: 3	2	6
Moran, [20] 1992, USA	14/7	39 36%	End-stage osteonecrosis of femoral head secondary to SCH after primary and revision THA	Revision	13 (86%) Posterior: 4% Lateral: 86% 11 (78.5%)	11/7 (157)	3 (42.85)	1 (14.28)	1 (14.28)	2 (25.57)	0	0	Resection: 1 Ileus: 1 Drainage: 1 Femoral perforation: 2 Nerve palsy: 1 DVT: 2, UTI: 2, Hematoma: 2, Sepsis: 1	5.3 (3.5–12.2)	5
Hickman, [18] 1997, USA	11/8	40 36.4%	Primary HA with SC	Revision	25%, Posterolateral, NA	5/8 (62.5)	1 (12.50)	1 (12.50)	0	1 (12.50)	1 (12.50)	0	Blood loss: 3	5 (2–12)	6
Bishop, [14] 1988, USA	11/4	30.6 46%	Confirmed SS, SC, or S-thalassemia, at Grady Hospital, primary THA in 1974–1984	Revision	2 (11.7%), Posterior or direct lateral, NA	2/4 (50)	0	0	1 (25)	1 (25)	0	0		2.7 (2.8–13) years	6
Kalacy, [32] 2007, Turkey	10/11	35 50%	SCD underwent revision THA in 1988–99	Revision	NA Anterolateral: 27.2% Posterior: 36.3% Standard trochanteric osteotomy: 18.1% Extended lateral trochanteric osteotomy: 18.1% 1 (10%)	0	5 (45.45)	2 (18.18)	2 (18.18)	3 (27.27)	0	2 (18.18)	Hematoma: 4, Osteolysis: 4 Wound drainage: 3 Super infection: 1	7.6 (2–13)	5

AVN: Avascular necrosis, Cement: Fully cemented technique, DVT: Deep vein thrombosis, GIT: Gastrointestinal tract complications, OA: Osteoarthritis, ONFH: Osteonecrosis of the femoral head, PE: Pulmonary embolism, PJI: Prosthetic joint infection, SCD: Sickle cell disease, SS%: Homozygous sickle cell gene (hemoglobin SS), THA: Total hip arthroplasty, UTI: Urinary tract infection. NA: Not applicable.

**Table 2:** Prosthetic joint infection-causative organisms.

Author, Year	Causative Organisms
Abdullah S. AlOmran, 2023 [22]	*Staphylococcus epidermidis*
Andrew R. Bishop, 1988 [14]	*Staphylococcus epidermidis* *(Corynebacterium)* *Staphylococcus aureus.*
Kalacy A, 2007 [32]	*Staphylococcus aureus* *Acinetobacter baumannii* *Klebsiella oxytoca* *Citrobacter freundii*
Acurio, 1992 [12]	*Staphylococcus epidermidis* *Staphylococcus aureus* *Acinetobacter calcoaceticus* *Proteus vulgaris* *Pseudomonas herrelea*
Sene, 2009 [29]	*Staphylococcus*
Michael C. Moran, 1992 [20]	*Staphylococcus epidermis* *Bacteroides fragilis then Enterococcus*
Ilyas I, 2017 [24]	*Staphylococcus Aureus* *Staphylococcus epidermidis*
Jack CM, 2016 [28]	*Pseudomonas*
Philippe Hernigou, 2008 [1]	*Staphylococcus aureus* *Acinetobacter* *Proteus* *Pseudomonas*

## Data Availability

Data used in this study are available on request.

## Supplementary Materials

The following supporting information can be downloaded at: https://www.mdpi.com/article/10.3390/jcm13144129/s1, Supplementary S1: Postoperative complications after hip arthroplasty in sickle cell disease patients with different arthroplasty approaches: a systematic review and network meta-analysis. Supplementary S2: Figures S1–S3.

## List of Abbreviations

THA	Total hip arthroplasty
SCD	Sickle cell disease
PJIs	Prosthetic joint infections
AVN	Avascular necrosis
DVT	Deep vein thrombosis
GIT	Gastrointestinal tract complications
OA	Osteoarthritis
ONFH	Osteonecrosis of the femoral head
PE	Pulmonary embolism
HbSS	Homozygous sickle cell gene (hemoglobin SS)
UTIs	Urinary tract infections
