# Supplementary material for: Total Hip Arthroplasty Complications in Sickle Cell Disease: Systematic Review and Meta-Analysis
Source: J Clin Med. 2024 Jul 15;13(14):4129. doi: 10.3390/jcm13144129 (PMC11277652; doi:10.3390/jcm13144129)
Supplement: Supplementary file 1 [file jcm-13-04129-s001.zip › jcm-3075084-Supplementary S1.pdf]

Postoperative complications after hip arthroplasty in sickle cell disease patients with different arthroplasty approaches: a systematic review and network meta-analysis

Mesh term	Text word
"Postoperative Complications"	Complication, Postoperative<br><br>Complications, Postoperative<br><br>Postoperative Complication

"Hip arthroplasty"	Arthroplasties, Replacement, Hip<br><br>Arthroplasty, Hip Replacement<br><br>Hip Replacement Arthroplasties<br><br>Hip Prosthesis Implantation<br><br>Hip Prosthesis Implantations<br><br>Implantation, Hip Prosthesis<br><br>Prosthesis Implantation, Hip<br><br>Replacement Arthroplasties, Hip<br><br>Replacement Arthroplasty, Hip<br><br>Arthroplasties, Hip Replacement<br><br>Hip Replacement Arthroplasty<br><br>Hip Replacement, Total<br><br>Replacement, Total Hip<br><br>Total Hip Replacements<br><br>Total Hip Replacement<br><br>Total Hip Arthroplasty<br><br>Arthroplasty, Total Hip<br><br>Hip Arthroplasty, Total<br><br>Total Hip Arthroplasties

**“sickle cell”**	Cell Trait, Sickle<br><br>Cell Traits, Sickle<br><br>Sickle Cell Traits<br><br>Trait, Sickle Cell<br><br>Traits, Sickle Cell<br><br>• Anemias, Sickle Cell<br>• Sickle Cell Anemias<br>• Hemoglobin S Disease<br>• Disease, Hemoglobin S<br>• Hemoglobin S Diseases<br>• Sickle Cell Anemia<br>• Sickle Cell Disorders<br>• Cell Disorder, Sickle<br>• Cell Disorders, Sickle<br>• Sickle Cell Disorder<br>• Sickling Disorder Due to Hemoglobin S<br>• HbS Disease<br>• Sickle Cell Disease<br>• Cell Disease, Sickle<br>• Cell Diseases, Sickle<br>• Sickle Cell Diseases<br>• Disease, Hemoglobin SC<br>• Disease, SC<br>• SC Diseases<br>• Sickle Cell Hemoglobin C Disease<br>• Hemoglobin SC Diseases

Database	Search strategy	No. of articles	Author name
MEDLINE central	Search **((((((((((((((((((((((Hip arthroplasty[MeSH Terms]) OR Arthroplasties, Replacement, Hip[Text Word]) OR Arthroplasty, Hip Replacement[Text Word]) OR Hip Replacement Arthroplasties[Text Word]) OR Hip Prosthesis Implantation[Text Word]) OR Hip Prosthesis Implantations[Text Word]) OR Implantation, Hip Prosthesis[Text Word]) OR Prosthesis Implantation, Hip[Text Word]) OR Replacement Arthroplasties, Hip[Text Word]) OR Replacement Arthroplasty, Hip[Text Word]) OR Arthroplasties, Hip Replacement[Text Word]) OR Hip Replacement Arthroplasty[Text Word]) OR Hip Replacement, Total[Text Word]) OR Replacement, Total Hip[Text Word]) OR Total Hip Replacements[Text Word]) OR Total Hip Replacement[Text Word]) OR Total Hip Arthroplasty[Text Word]) OR Arthroplasty, Total Hip[Text Word]) OR Hip Arthroplasty, Total[Text Word]) OR Total Hip Arthroplasties[Text Word])) AND (((((((((((((((((((sickle cell[MeSH Terms]) OR Anemias, Sickle Cell[Text Word]) OR Sickle Cell Anemias[Text Word]) OR Hemoglobin S Disease[Text Word]) OR Disease, Hemoglobin S[Text Word]) OR Hemoglobin S Diseases[Text Word]) OR Sickle Cell Anemia[Text Word]) OR Sickle Cell Disorders[Text Word]) OR Cell Disorder*, Sickle[Text Word]) OR Sickle Cell Disorder[Text Word]) OR Sickling Disorder Due to Hemoglobin S[Text Word]) OR HbS Disease[Text Word]) OR Sickle Cell Disease[Text Word]) OR Cell Disease*, Sickle[Text Word]) OR Sickle Cell Diseases[Text Word]) OR Disease, Hemoglobin SC[Text Word]) OR Disease, SC[Text Word]) OR Sickle Cell Hemoglobin C Disease[Text Word]) OR Hemoglobin SC Diseases[Text Word])) AND ((((“Postoperative Complications”[MeSH Terms]) OR**	61	Dr. shaimaa

	**Complication, Postoperative[Text Word]) OR Complications, Postoperative[Text Word]) OR Postoperative Complication[Text Word])**		
PubMed	Search (((((((((((((((((((("Anemias, Sickle Cell"[Text Word]) OR "Sickle Cell Anemias"[Text Word]) OR "Hemoglobin S Disease"[Text Word]) OR "Disease, Hemoglobin S"[Text Word]) OR "Hemoglobin S Diseases"[Text Word]) OR "Sickle Cell Anemia"[Text Word]) OR "Sickle Cell Disorders"[Text Word]) OR "Cell Disorder, Sickle"[Text Word]) OR "Cell Disorders, Sickle"[Text Word]) OR "Sickle Cell Disorder"[Text Word]) OR "Sickling Disorder Due to Hemoglobin S"[Text Word]) OR "HbS Disease"[Text Word]) OR "Sickle Cell Disease"[Text Word]) OR "Cell Disease, Sickle"[Text Word]) OR "Cell Diseases, Sickle"[Text Word]) OR "Sickle Cell Diseases"[Text Word])) OR Anemia, Sickle Cell[MeSH Terms])) AND (("Arthroplasty, Replacement, Hip"[MeSH Terms]) OR ((((((((((((((((((("Arthroplasties, Replacement, Hip"[Text Word]) OR "Arthroplasty, Hip Replacement"[Text Word]) OR "Arthroplasty, Hip Replacement"[Text Word]) OR "Hip Prosthesis Implantation"[Text Word]) OR "Hip Prosthesis Implantations"[Text Word]) OR "Implantation, Hip Prosthesis"[Text Word]) OR "Prosthesis Implantation, Hip"[Text Word]) OR "Replacement Arthroplasties, Hip"[Text Word]) OR "Replacement Arthroplasty, Hip"[Text Word]) OR "Arthroplasties, Hip Replacement"[Text Word]) OR "Hip Replacement Arthroplasty"[Text Word]) OR "Hip Replacement, Total"[Text Word]) OR "Replacement, Total Hip"[Text Word]) OR "Total Hip Replacements"[Text Word]) OR "Total Hip Replacement"[Text Word]) OR "Total Hip Arthroplasty"[Text Word]) OR "Arthroplasty, Total Hip"[Text Word]) OR "Hip Arthroplasty, Total"[Text Word]) OR "Total Hip Arthroplasties"[Text Word]))) AND	61	Dr.Esraa

	((((("Complication, Postoperative"[Text Word]) OR "Complications, Postoperative"[Text Word]) OR "Postoperative Complication"[Text Word])) OR "Postoperative Complications"[MeSH Terms])		
scopus	(arthroplasties, AND replacement, AND hip OR arthroplasty, AND hip AND replacement OR hip AND replacement AND arthroplasties OR hip AND prosthesis AND implantation OR hip AND prosthesis AND implantations OR implantation, AND hip AND prosthesis OR prosthesis AND implantation, AND hip OR replacement AND arthroplasties, AND hip OR replacement AND arthroplasty, AND hip OR arthroplasties, AND hip AND replacement OR hip AND replacement AND arthroplasty OR hip AND replacement, AND total OR replacement, AND total AND hip OR total AND hip AND replacements OR total AND hip AND replacement OR total AND hip AND arthroplasty OR arthroplasty, AND total AND hip OR hip AND arthroplasty, AND total OR total AND hip AND arthroplasties) AND (postoperative AND complication) AND (sickle AND cell AND diseases OR cell AND diseases, AND sickle OR cell AND disease, AND sickle OR sickle AND cell AND disease OR hbs AND disease OR sickling AND disorder AND due AND to AND hemoglobin AND s OR sickle AND cell AND disorder OR cell AND disorders, AND sickle OR cell AND disorder, AND sickle OR sickle AND cell AND disorders OR sickle AND cell AND anemia OR hemoglobin AND s AND diseases OR disease, AND hemoglobin AND s OR hemoglobin AND s AND disease OR sickle AND cell AND anemias OR anemias, AND sickle AND cell)	13	Dr.Esraa
sage	"Anemias, Sickle Cell" OR "Sickle Cell Anemias" OR "Hemoglobin S Disease" OR "Disease, Hemoglobin S" OR	39	Dr.Esraa

	"Hemoglobin S Diseases" OR "Sickle Cell Anemia" OR "Sickle Cell Disorders" OR "Cell Disorder, Sickle" OR "Cell Disorders, Sickle" OR "Sickle Cell Disorder" OR "Sickling Disorder Due to Hemoglobin S" OR "HbS Disease" OR "Sickle Cell Disease" OR "Cell Disease, Sickle" OR "Cell Diseases, Sickle" OR "Sickle Cell Diseases" AND "postoperative complication" OR " Postoperative complications" OR "Complications, Postoperative" AND "Arthroplasties, Replacement, Hip" OR "Arthroplasty, Hip Replacement" OR "Hip Replacement Arthroplasties" OR "Hip Prosthesis Implantation" OR "Hip Prosthesis Implantations" OR "Implantation, Hip Prosthesis" OR "Prosthesis Implantation, Hip" OR "Replacement Arthroplasties, Hip" OR "Replacement Arthroplasty, Hip" OR "Arthroplasties, Hip Replacement" OR "Hip Replacement Arthroplasty" OR "Hip Replacement, Total" OR "Replacement, Total Hip" OR "Total Hip Replacements" OR "Total Hip Replacement" OR "Total Hip Arthroplasty" OR "Arthroplasty, Total Hip" OR "Hip Arthroplasty, Total" OR "Total Hip Arthroplasties"		
Embase			
ProQuest	abstract(Arthroplasties, Replacement, Hip<br>) OR abstract(Arthroplasty, Hip Replacement<br>) OR abstract(Hip Prosthesis Implantation<br>) OR abstract(Replacement Arthroplasty, Hip<br>) OR abstract(Total Hip Replacement*<br>) OR abstract(Total Hip Arthroplasty<br>) AND abstract(Sickle Cell Anemias) OR abstract(Sickle Cell Diseases<br>) OR abstract(Cell Disease, Sickle<br>) AND abstract(Postoperative Complication*<br>)	1651	shaimaa
SciELO	(((((((((((TS=(Arthroplasty, Hip Replacement)) OR TS=(Hip Prosthesis Implantation)) OR TS=(Prosthesis Implantation, Hip)) OR TS=(Hip Replacement Arthroplasty)) OR	10	Dr.shaimaa

	TS=(Total Hip Replacement*)) OR TS=(Total Hip Arthroplasties)) AND TS=(Anemias, Sickle Cell)) OR TS=(Hemoglobin S Disease)) OR TS=(Cell Disorder, Sickle)) OR TS=(HbS Disease)) OR TS=(SC Diseases)) OR TS=(Hemoglobin SC Diseases)) AND TS=(postoperative complication*)		
Web of Science	(((((((((((ALL=(Arthroplasty, Hip Replacement)) OR ALL=(Hip Prosthesis Implantation)) OR ALL=(Prosthesis Implantation, Hip)) OR ALL=(Hip Replacement Arthroplasty)) OR ALL=(Total Hip Replacement*)) OR ALL=(Total Hip Arthroplasties)) AND ALL=(Anemias, Sickle Cell)) OR ALL=(Hemoglobin S Disease)) OR ALL=(Cell Disorder, Sickle)) OR ALL=(HbS Disease)) OR ALL=(SC Diseases)) OR ALL=(Hemoglobin SC Diseases)) AND ALL=(postoperative complication*)	**1315**	Dr.shaimaa
Google Scholar	"Total Hip Arthroplasty" OR "Hip Replacement Arthroplasty" OR "Hip Prosthesis Implantation" AND "Sickle Cell Anemia" OR "Sickle Cell Disorders" OR "Sickle Cell Disease"	80	Dr. shaimaa
total		3230	
duplicates		125 +28	
