# Supplementary material for: Total Hip Arthroplasty Complications in Sickle Cell Disease: Systematic Review and Meta-Analysis
Source: J Clin Med. 2024 Jul 15;13(14):4129. doi: 10.3390/jcm13144129 (PMC11277652; doi:10.3390/jcm13144129)

Figure S1 Funnel plots

Crisis

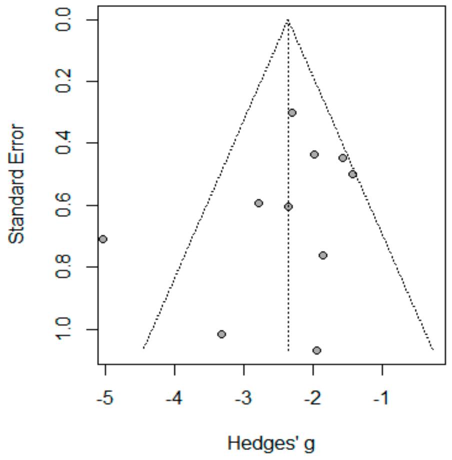

Dislocation

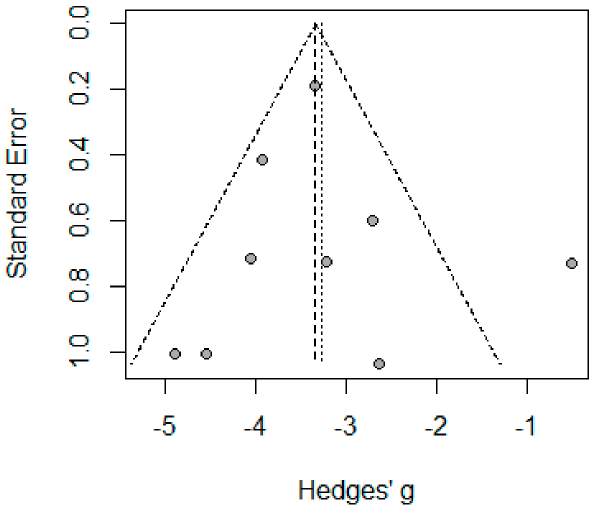

Aseptic loosening

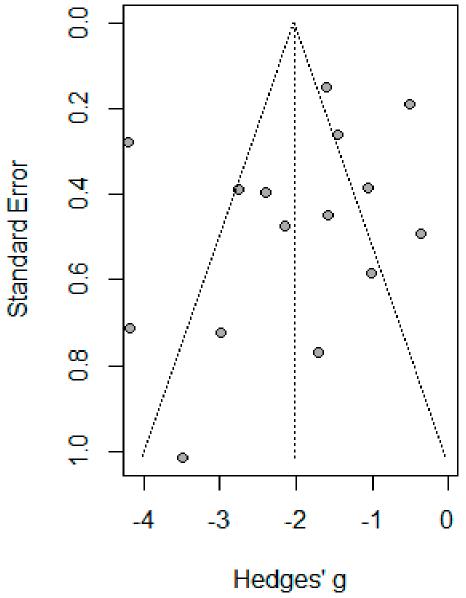

Ossification

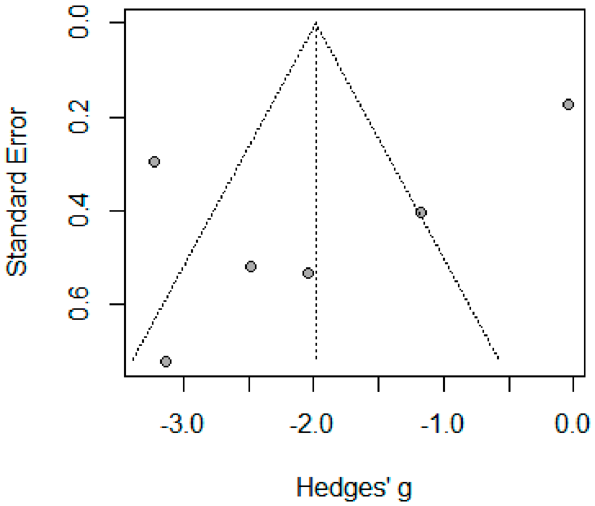

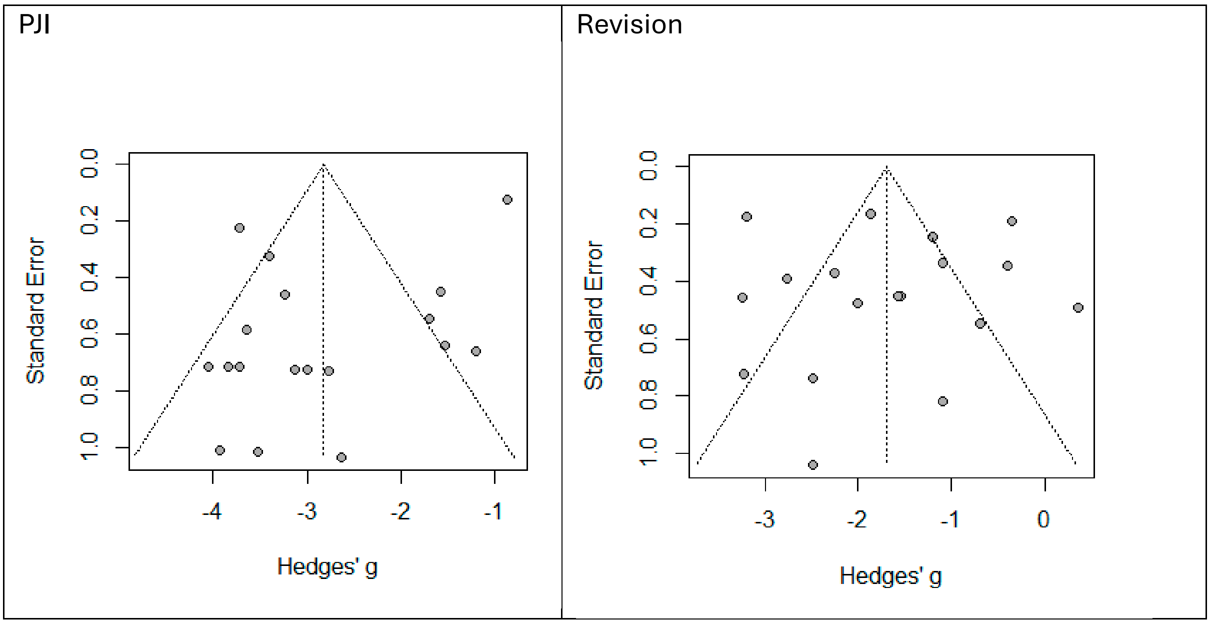
PJI
Standard Error
0.0
0.2
0.4
0.6
0.8
1.0
-4
-3
-2
-1
Hedges' g
Revision
Standard Error
0.0
0.2
0.4
0.6
0.8
1.0
-3
-2
-1
0
Hedges' g

Figure S2 Leave one out sensitivity analysis

Total complications

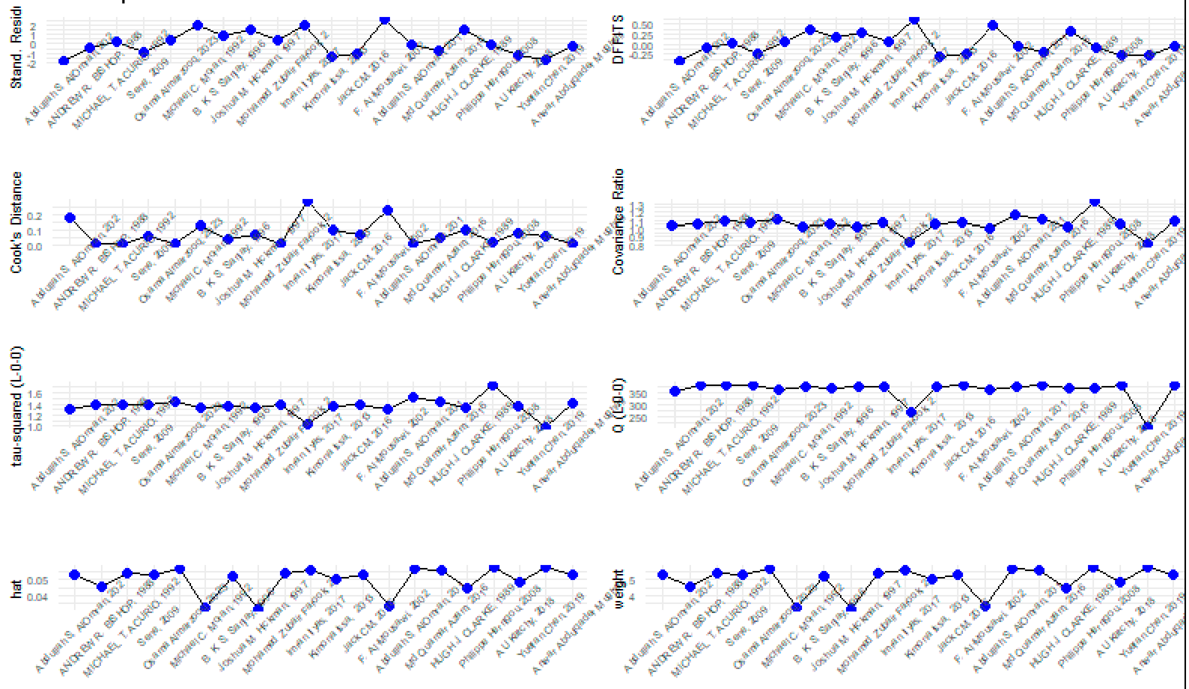

Crisis

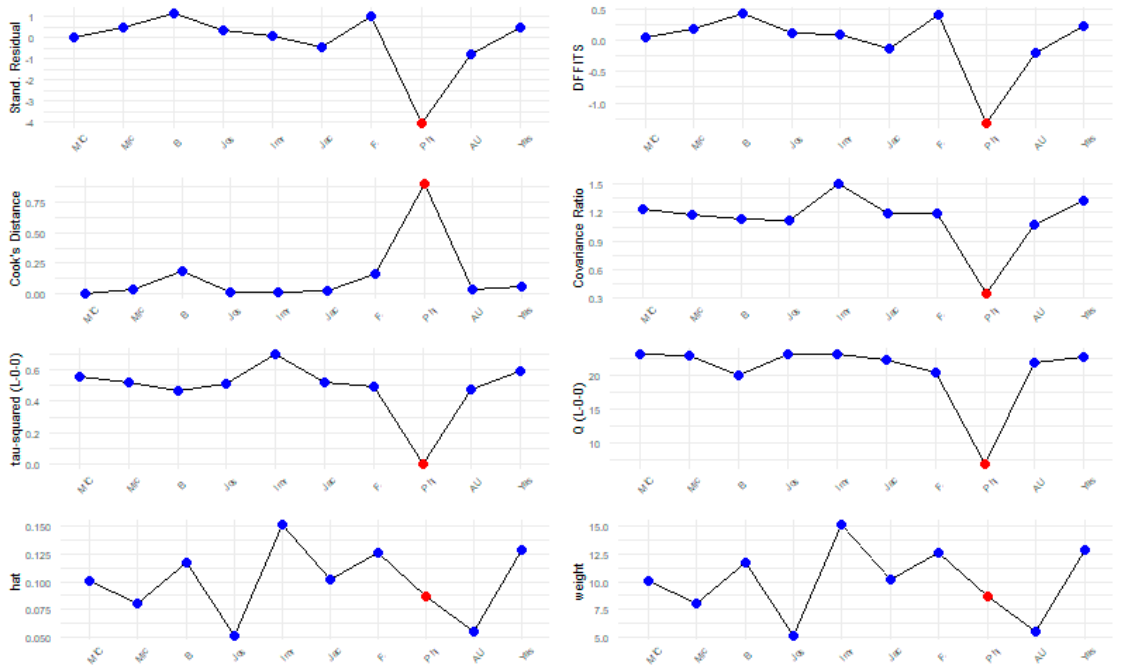

Dislocation

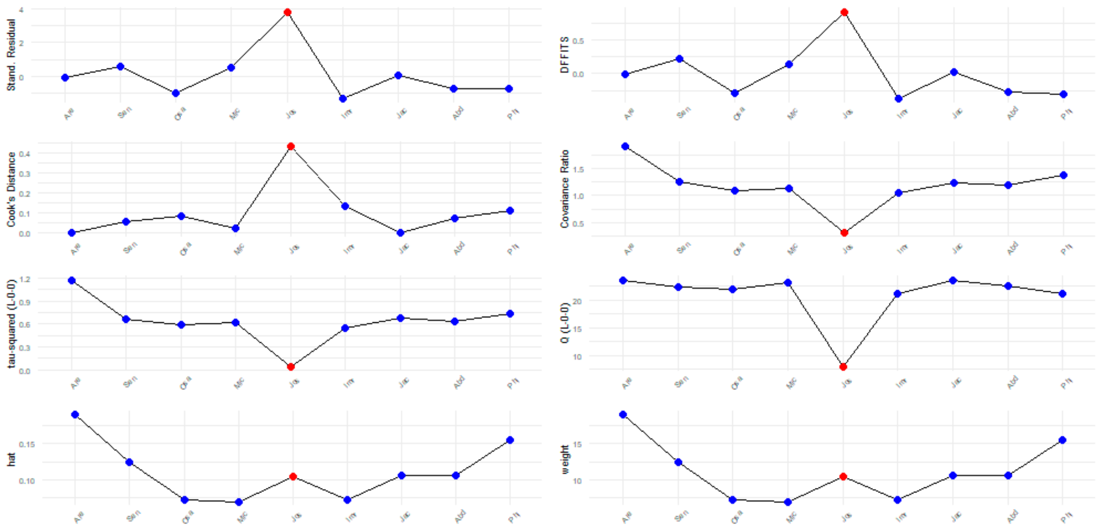

Aseptic loosening

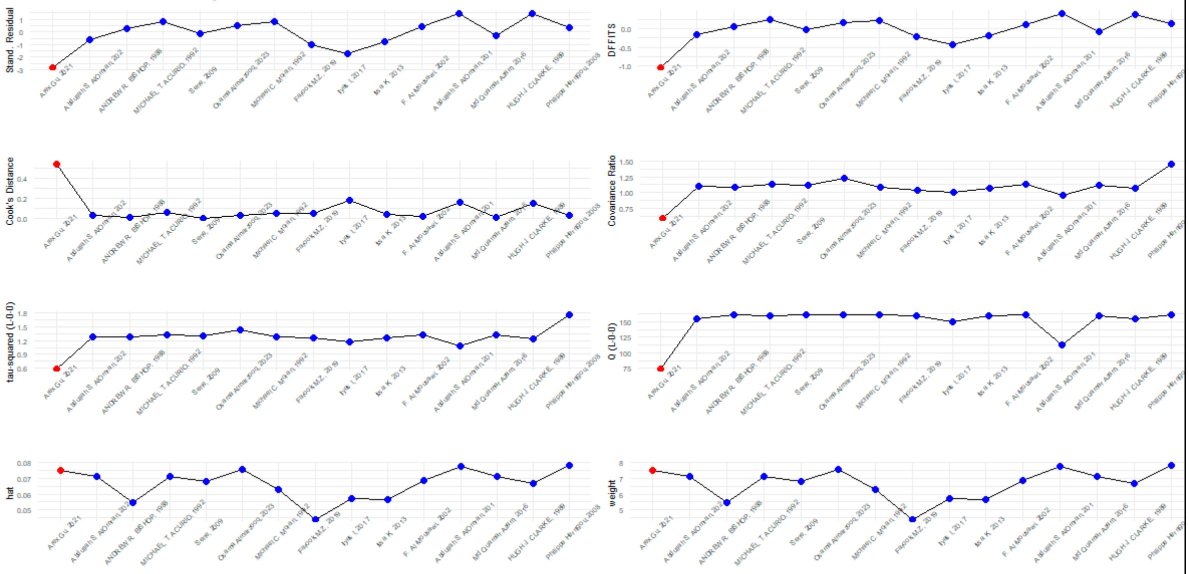

Ossification

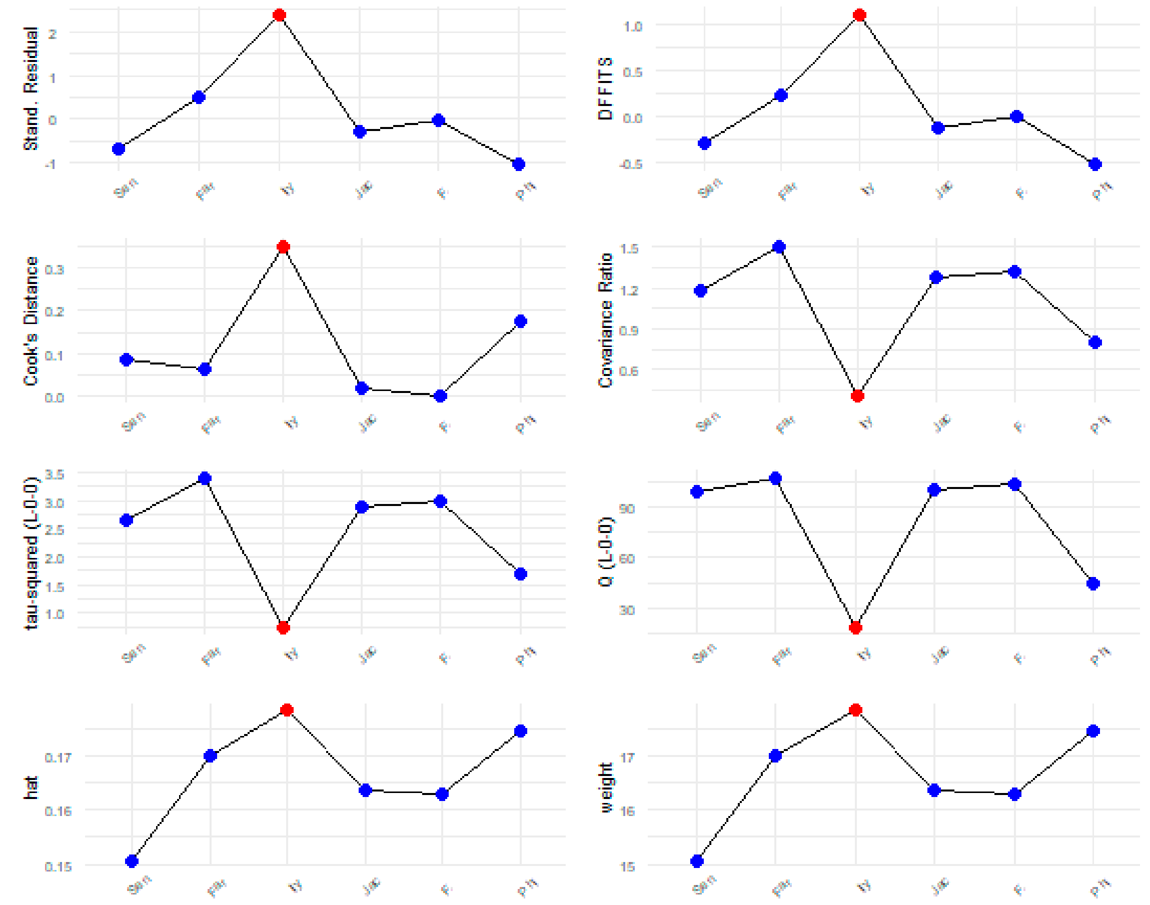
Stand. Residual
DFFITS
Cook's Distance
Covariance Ratio
tau-squared (L-0-0)
Q (L-0-0)
hat
weight

PJI

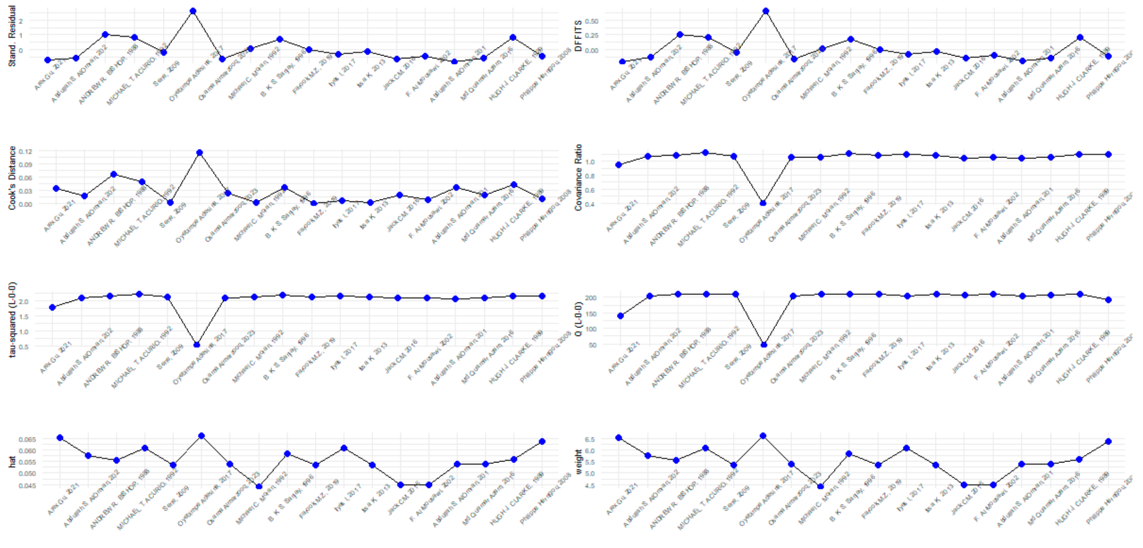
Stand. Residual
DFFITS
Cook's Distance
Covariance Ratio
tau-squared (L-0-0)
Q (L-0-0)
hat
weight

Revision

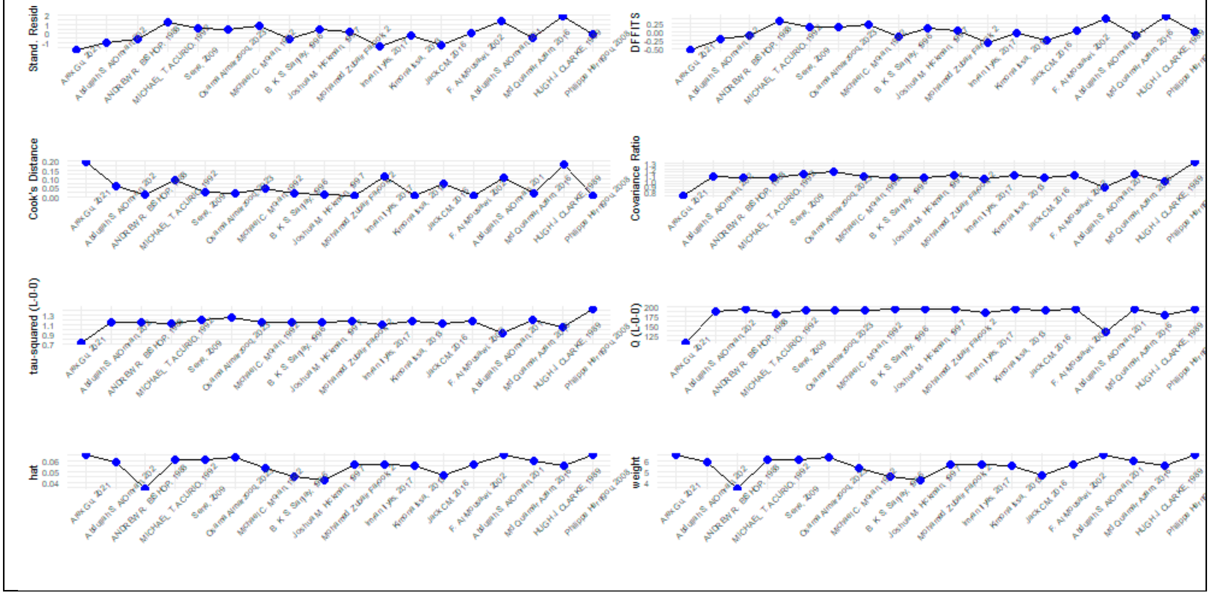
Stand. Resid
DFFITS
Cook's Distance
Covariance Ratio
tau-squared (L-0-0)
Q (L-0-0)
hat
weight

Figure S3 Forest plot after remove outliers or influential studies

Crisis

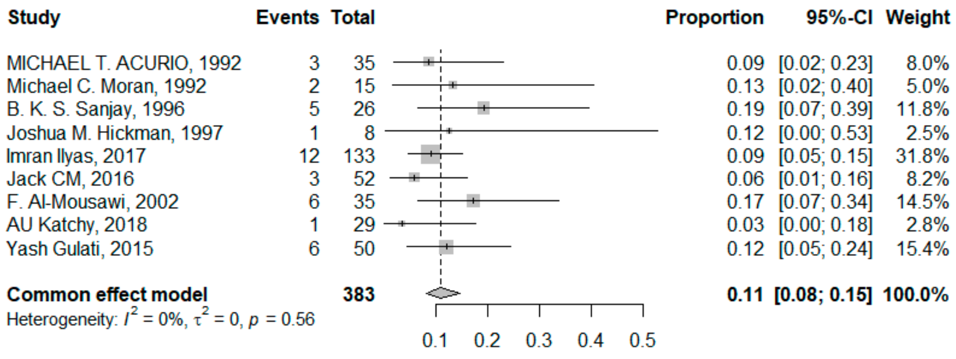

Dislocation

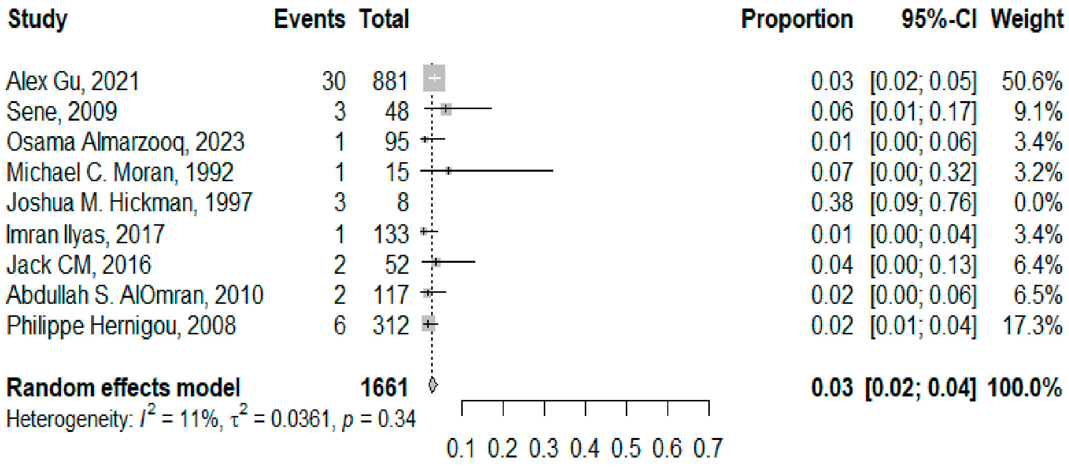

Aseptic loosening

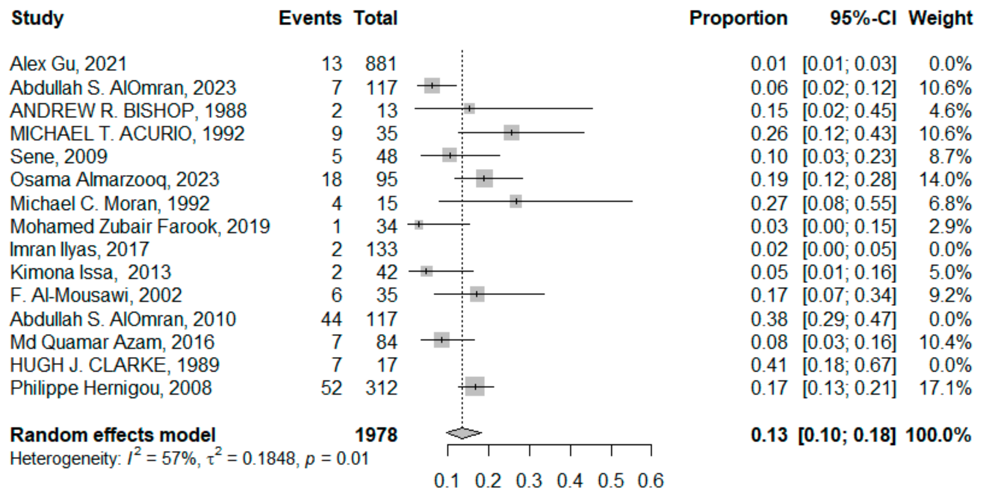

Ossification

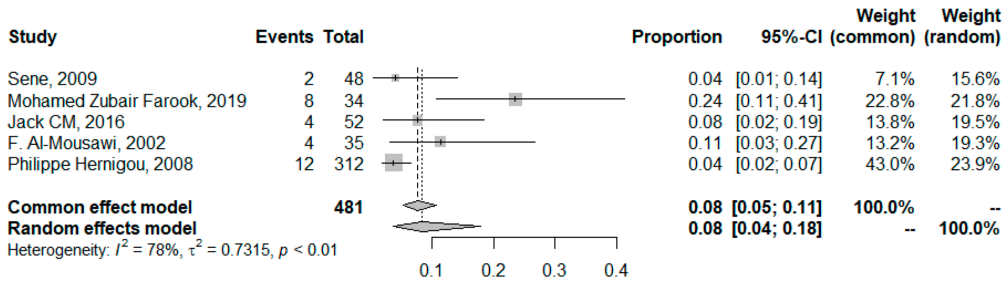

PJI

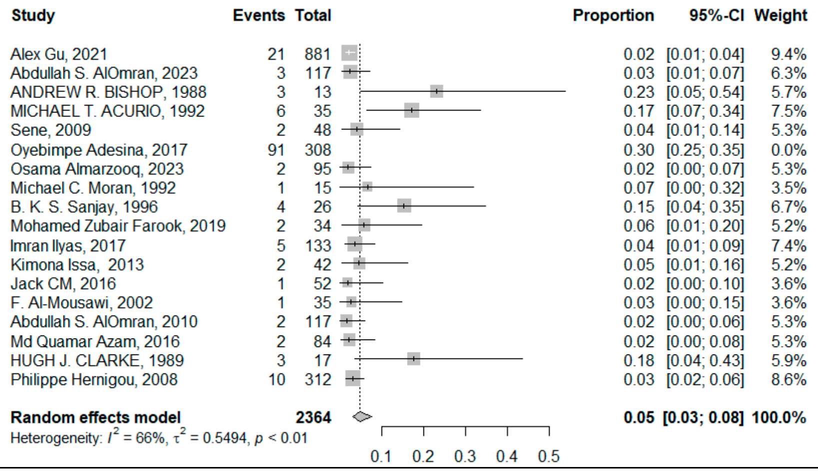

Revision

Study	Events	Total	Proportion	95%-CI	Weight
Alex Gu, 2021	35	881	0.04	[0.03; 0.05]	0.0%
Abdullah S. AlOmran, 2023	7	117	0.06	[0.02; 0.12]	9.1%
ANDREW R. BISHOP, 1988	1	13	0.08	[0.00; 0.36]	2.5%
MICHAEL T. ACURIO, 1992	14	35	0.40	[0.24; 0.58]	0.0%
Sene, 2009	12	48	0.25	[0.14; 0.40]	10.3%
Osama Almarzooq, 2023	22	95	0.23	[0.15; 0.33]	12.3%
Michael C. Moran, 1992	5	15	0.33	[0.12; 0.62]	6.4%
B. K. S. Sanjay, 1996	2	26	0.08	[0.01; 0.25]	4.3%
Joshua M. Hickman, 1997	2	8	0.25	[0.03; 0.65]	3.7%
Mohamed Zubair Farook, 2019	6	34	0.18	[0.07; 0.35]	8.0%
Imran Ilyas, 2017	5	133	0.04	[0.01; 0.09]	0.0%
Kimona Issa, 2013	5	42	0.12	[0.04; 0.26]	7.5%
Jack CM, 2016	2	52	0.04	[0.00; 0.13]	4.5%
F. Al-Mousawi, 2002	6	35	0.17	[0.07; 0.34]	8.0%
Abdullah S. AlOmran, 2010	48	117	0.41	[0.32; 0.50]	0.0%
Md Quamar Azam, 2016	8	84	0.10	[0.04; 0.18]	9.5%
HUGH J. CLARKE, 1989	10	17	0.59	[0.33; 0.82]	0.0%
Philippe Hernigou, 2008	42	312	0.13	[0.10; 0.18]	14.0%
**Random effects model**		**2064**	**0.14**	**[0.11; 0.19]**	**100.0%**

Heterogeneity: $I^2 = 58\%$, $\tau^2 = 0.2031$, $p < 0.01$

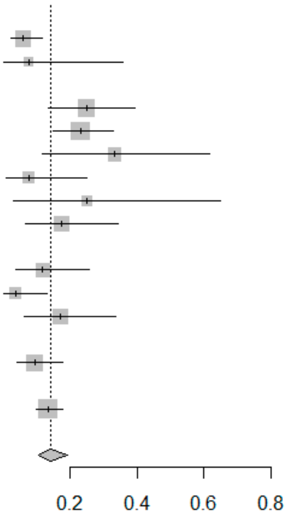

Supplement: Supplementary file 1 [file jcm-13-04129-s001.zip › jcm-3075084-Supplementary S2.pdf]
